# Constitutively Activated NLRP3 Inflammasome Causes Inflammation and Abnormal Skeletal Development in Mice

**DOI:** 10.1371/journal.pone.0035979

**Published:** 2012-04-27

**Authors:** Sheri L. Bonar, Susannah D. Brydges, James L. Mueller, Matthew D. McGeough, Carla Pena, Debbie Chen, Susan K. Grimston, Cynthia L. Hickman-Brecks, Soumya Ravindran, Audrey McAlinden, Deborah V. Novack, Daniel L. Kastner, Roberto Civitelli, Hal M. Hoffman, Gabriel Mbalaviele

**Affiliations:** 1 Division of Bone and Mineral Diseases, Washington University School of Medicine, St. Louis, Missouri, United States of America; 2 Division of Allergy, Immunology, and Rheumatology and Departments of Pediatrics and Medicine, University of California San Diego, La Jolla, California, United States of America; 3 Department of Orthopaedic Surgery, Washington University School of Medicine, St. Louis, Missouri, United States of America; 4 Medical Genetics Branch, Department of Health and Human Services/National Institutes of Health/National Health Genome Research Institute, Bethesda, Maryland, United States of America; French National Centre for Scientific Research, France

## Abstract

The NLRP3 inflammasome complex is responsible for maturation of the pro-inflammatory cytokine, IL-1β. Mutations in *NLRP3* are responsible for the cryopyrinopathies, a spectrum of conditions including neonatal-onset multisystem inflammatory disease (NOMID). While excessive production of IL-1β and systemic inflammation are common to all cryopyrinopathy disorders, skeletal abnormalities, prominently in the knees, and low bone mass are unique features of patients with NOMID. To gain insights into the mechanisms underlying skeletal abnormalities in NOMID, we generated knock-in mice globally expressing the D301N NLRP3 mutation (ortholog of D303N in human NLRP3). NOMID mice exhibit neutrophilia in blood and many tissues, including knee joints, and high levels of serum inflammatory mediators. They also exhibit growth retardation and severe postnatal osteopenia stemming at least in part from abnormally accelerated bone resorption, attended by increased osteoclastogenesis. Histologic analysis of knee joints revealed abnormal growth plates, with loss of chondrocytes and growth arrest in the central region of the epiphyses. Most strikingly, a tissue “spike" was observed in the mid-region of the growth plate in the long bones of all NOMID mice that may be the precursor to more severe deformations analogous to those observed in NOMID patients. These findings provide direct evidence linking a NOMID-associated NLRP3-activating mutation to abnormalities of postnatal skeletal growth and bone remodeling.

## Introduction

NLRP3, also called cryopyrin, is one of the most studied members of the NOD-like receptor (NLR) family, which are intracellular proteins involved in the initiation of the innate immune response. NLRP3 is capable of forming an inflammasome [1], [2], an intracellular protein complex responsible for activation of caspase 1 and the subsequent processing of pro-IL-1β and pro-IL-18 into mature IL-1β and IL-18, respectively. The NLRP3 inflammasome is activated by multiple danger-associated moieties, including ATP, glucose, monosodium urate, calcium pyrophosphate dihydrate and cholesterol crystals [3]–[5]. Dysregulated activation of this inflammasome is believed to be involved in the pathogenesis of various inflammatory and metabolic diseases such as gout, pseudogout, type-2 diabetes and atherosclerosis [3], [5]–[7].

Approximately 80 pathogenic mutations in the *NLRP3* gene have been identified in patients with systemic autoinflammatory disorders known as cryopyrinopathies or cryopyrin-associated periodic syndromes (CAPS), which include neonatal-onset multisystem inflammatory disease (NOMID), Muckle-Wells syndrome (MWS) and familial cold autoinflammatory syndrome (FCAS) [8]. *NLRP3* mutations are thought to cause constitutive inflammasome activation with some degree of genotype-phenotype correlation [9]. Each of the CAPS phenotypes is associated with excessive cytokine production, recurrent or chronic fever, urticaria-like rash, and joint and CNS symptoms. However, skeletal malformations are unique features of NOMID [10], [11], and a report on a cohort of NOMID patients revealed that the majority of these patients have bone deformities and/or are osteoporotic. Abnormal endochondral ossification was suspected in these patients [12].

Intramembranous and endochondral ossifications are two processes that with few exceptions, govern the development of flat and long bones, respectively. In the former process mesenchymal condensations form bone directly, whereas in the latter, they differentiate into chondrocytes which form the cartilage template for bone development [13]. After development, balanced bone formation and resorption ensures bone homeostasis. While unsynchronized events during development cause bone malformations, excessive bone resorption leads to bone loss. This loss occurs in a variety of pathological conditions in which the production of pro-inflammatory cytokines is increased, including postmenopausal osteoporosis, inflammatory diseases such as rheumatoid arthritis and aseptic implant loosening, and infectious diseases including endotoxemia and periodontitis [14], [15]. Cytokines such as IL-1β, IL-6 and tumor necrosis factor-α (TNF-α) have dual negative effects on bone health as they inhibit bone formation and enhance bone resorption [16], [17]. While bone formation is the function of osteoblasts (OB), bone resorption is the main function of osteoclasts (OC), hematopoietic cells of the monocyte/macrophage lineage [18]. Differentiation, survival and activity of OC depend upon the expression of RANKL, a TNF family member [18]. Pro-inflammatory cytokines regulate both RANKL expression and act in synergy with this factor to propagate inflammation-associated bone erosion [19]. Since high serum and tissue levels of IL-1β and IL-6 are characteristic of CAPS, it is reasonable to hypothesize that inflammatory bone loss occurs in this autoinflammatory disease spectrum and perhaps other NLRP3-associated disorders.

To model the human NOMID syndrome and to achieve insights into its associated skeletal abnormalities, we generated mice globally expressing the D301N mutation in *Nlrp3*, corresponding to the D303N mutation in human *NLRP3*, linked to NOMID. NOMID mice phenocopy several features of human NOMID such as early onset systemic inflammation and growth retardation. These mice also have disorganized growth plates and low bone mass associated with exuberant osteoclastogenesis, suggesting a strong link between this NOMID associated mutation and abnormal skeletal development.

## Results

### NOMID mice exhibit systemic inflammation and reduced survival

The aspartate 303 to asparagine (D303N) substitution has been identified in NOMID and severe MWS patients [10], [11]. This autosomal dominant point mutation occurs near the Mg^2+^ binding site in the NLRP3 NACHT domain and is thought to cause a conformational change that confers ligand-independent constitutive activation of the mutated NLRP3 inflammasome. To further understand the pathological impact of this mutation, we generated knock-in mice expressing D301N NLRP3 (D303N ortholog of human NLRP3) using a previously described strategy [20]. Due to the presence of an intronic FLOXed neomycin resistance cassette, the expression of the mutation does not occur unless knock-in mice are first bred with mice expressing Cre recombinase (Fig. S1). Zona pelucida 3-Cre was used to induce global expression of D301N NLRP3 similar to that observed in NOMID patients.

**Figure 1:**
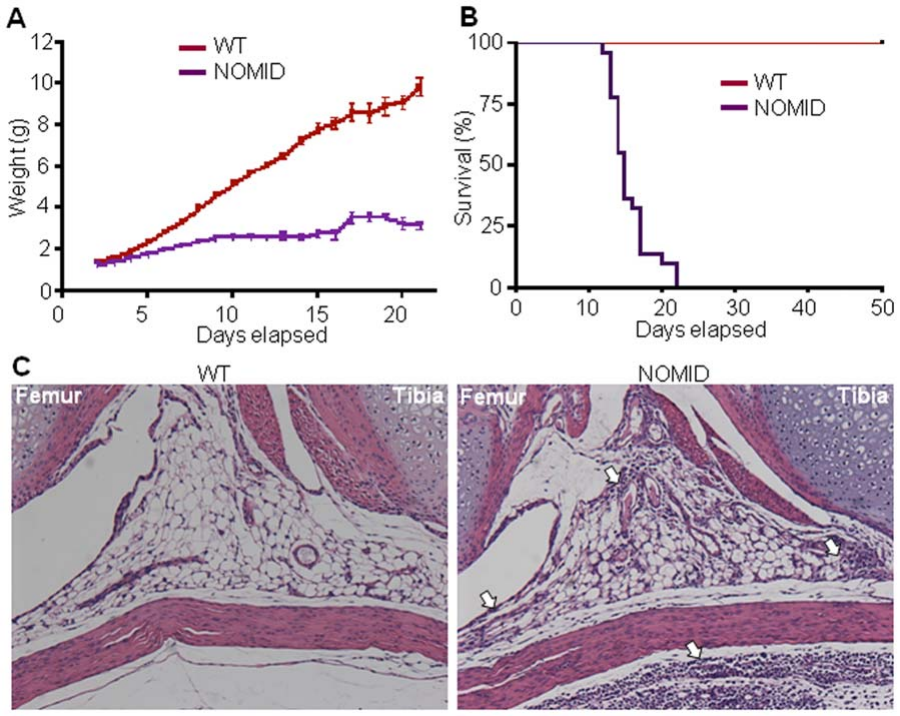
NOMID mice exhibit growth retardation, perinatal death and inflammation in the joints. Body weight (*A*) and survival (*B*) were monitored daily for 3 weeks (22–25 mice/genotype). NOMID mice demonstrated significantly reduced body weight compared to WT mice by day 5, and most died by 2 weeks of age. (*C*) H&E staining of the knee joints from P8 mice. Original magnification, ×10. NOMID mice displayed massive leukocytic infiltrates in the joints and surrounding tissues (arrows).

**Figure 2:**
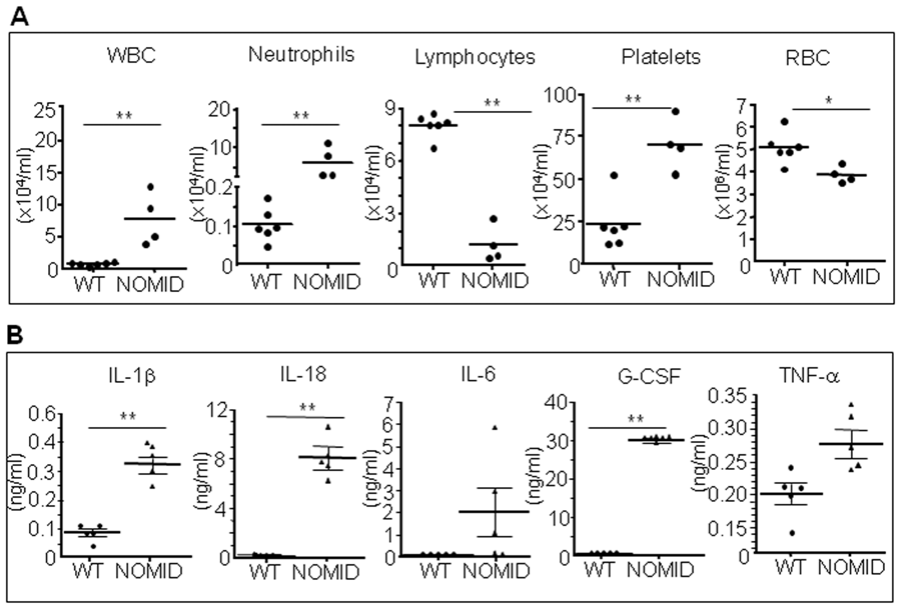
NOMID mice develop leukocytosis associated with high levels of inflammatory mediators. Complete blood cell counts (*A*) and serum cytokine analysis (*B*) were carried out on samples harvested from P12–13 mice (n = 4–7). NOMID mice developed neutrophilia, lymphopenia, thrombocytosis and anemia and produced higher levels of several inflammatory mediators. G-CSF values were extrapolated beyond the standard range. IL-6 and TNF-α levels were near statistical significance between genotypes. Data are expressed as mean ± S.E.M. *P<0.05, **P<0.007. WBC, white blood cells; RBC, red blood cells.

NOMID pups were born at the expected Mendelian frequency. Although often indistinguishable from wild-type (WT) siblings at birth, NOMID mice exhibited growth retardation and significantly lower body weight obvious by post birth day 5 (P5) (Fig. 1A), and the mice usually died by 2–3 weeks of age (Fig. 1B). Body fat mass measured at P13 by dual-energy X-ray absorptiometry (DXA) was not significantly different between NOMID and WT mice (21.6±0.5% vs. 20.6±0.5%, respectively; p>0.10, n = 5). Haematoxylin and eosin (H&E) staining showed granulocytic infiltrates in various tissues, including joints (Fig. 1C) and meninges (Fig. S2). Complete blood cell counts further revealed that NOMID mice exhibited peripheral neutrophilic leukocytosis, with accompanying thrombocytosis, severe lymphopenia and anemia (Fig. 2A). This phenotype is reminiscent of other models of *Nlrp3* variants [20], [21], and more importantly, of CAPS patients.

To gain further insights into the phenotype of NOMID mice, we measured the serum levels of inflammatory mediators. Serum IL-1β and IL-18 as well as the granulocyte growth factor, G-CSF, were significantly increased in serum from NOMID mice compared to WT mice (Fig. 2B). Serum levels of IL-3, IL-4, IL-6, IL-9, IL-13, GM-CSF, IFN-γ, TNF-α and several chemokines (e.g., Eotaxin, KC, MCP-1, MIP-1α, MIP-1β and RANTES) were also higher in NOMID mice (Fig. 2B and Fig. S3). In contrast, cytokines of activated T cells such as IL-2, IL-10 and IL-17 were not up-regulated in NOMID mice. These findings are in agreement with our previous results [20] and the notion that CAPS are primarily disorders of the innate immune system. While IL-1α levels were also not significantly different between genotypes, IL-5 and IL-12(p40) levels were lower in NOMID mice (Fig. S3). The significance of this finding is not clear. Thus, NOMID mice exhibit the systemic inflammation, hematologic features, cytokine levels, and poor growth characteristic of the human disease, validating the use of this model in further understanding the pathological consequences of NOMID.

### NOMID mice exhibit stunted skeletal growth, disorganized growth plates and reduced bone mass

**Figure 3:**
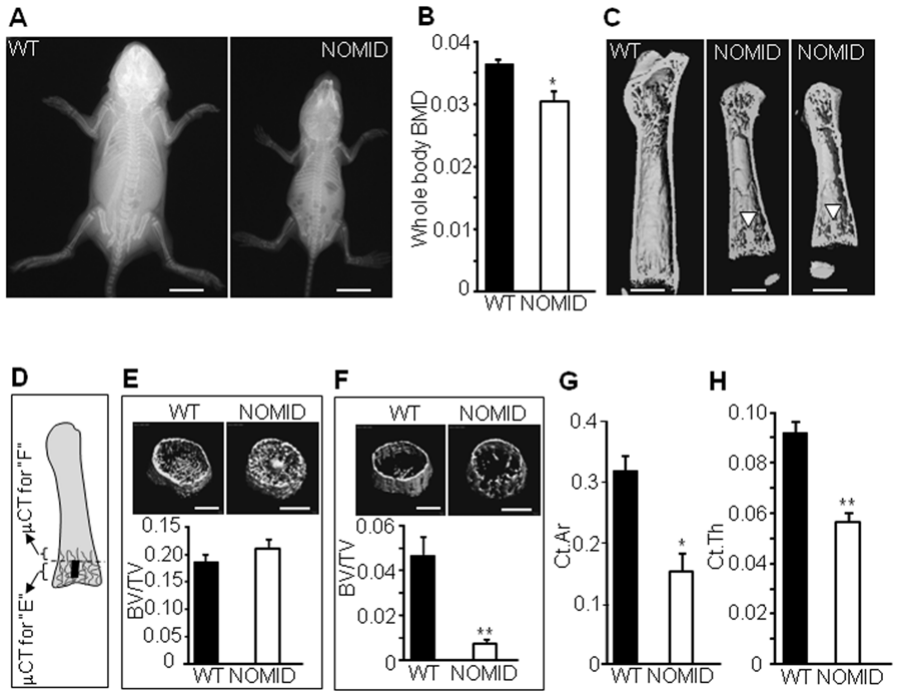
NOMID mice exhibit stunted skeletal growth and reduced bone mass. X-ray radiography (*A*), DXA (*B*) or μCT analysis of the femur (*C, E*–*H*) of NOMID and WT mice at age P13. DXA and μCT 3D reconstruction revealed generalized osteopenia and the presence of a tissue spike across the growth plate (*C*, arrowhead) in 2 NOMID mice. (*D*) The metaphyseal region containing (*E*) or contiguous (*F*) to the spike (depicted in black in Fig. 3D) where trabecular bone volume (BV/TV) was quantified. BV/TV was unchanged in the metaphyseal region that included the spike, but was decreased in the region contiguous to this structure. NOMID mice also exhibited significantly lower cortical area (*G*) and thinner cortical bone (*H*). Quantitative data were obtained from 5–6 mice/genotype and expressed as the mean ± S.E.M. *P<0.05; **P<0.007. BMD, bone mineral density.

NOMID-associated *NLRP3* mutations cause skeletal malformations and low bone mass not generally observed in MWS or FCAS [10], [11]. We therefore asked whether the massive leukocytic infiltration noted in NOMID mouse joints (Fig. 1C) might be associated with a bone phenotype similar to that observed in patients. By high resolution radiography, the skeleton of NOMID mice at age P13 was markedly smaller than WT littermates (Fig. 3A). Importantly, NOMID mice were significantly osteopenic relative to WT mice based on DXA analysis of whole body bone mineral density (BMD), which includes cortical and trabecular compartments of the axial and appendicular skeleton (Fig. 3B). Furthermore, 3D reconstruction of bone architecture by microcomputed tomography (μCT) demonstrated decreased femur size in NOMID relative to WT mice (Fig. 3C).

We also observed the development of a tissue “spike" across the mid region of the growth plate in NOMID mice (Fig. 3C, arrowhead). The tissue spike had an average length of 473.6±102.6 µm at P13 and was approximately 8.6% of the average length of the bone, occurring in the femurs and tibiae, prominent sites of bone deformities in NOMID patients [10], [12]. We therefore carried out structural analyses in the metaphyseal region that included or was contiguous to the spike (Fig. 3D). Despite the low whole body BMD, there was abundant trabeculation in the metaphyseal region around the spike in femurs of NOMID mice, with no difference in trabecular bone volume (BV/TV) between the genotypes (Fig. 3E and Fig. S4A, S4C and S4E). In contrast, trabecular bone mass in the region contiguous to the spike area was >80% reduced in NOMID mice compared to control littermates (Fig. 3F and Fig. S4B, S4D and S4F). Remarkably, NOMID mice also exhibited significantly lower cortical area and thickness relative to WT mice (Fig. 3G and 3H).

**Figure 4:**
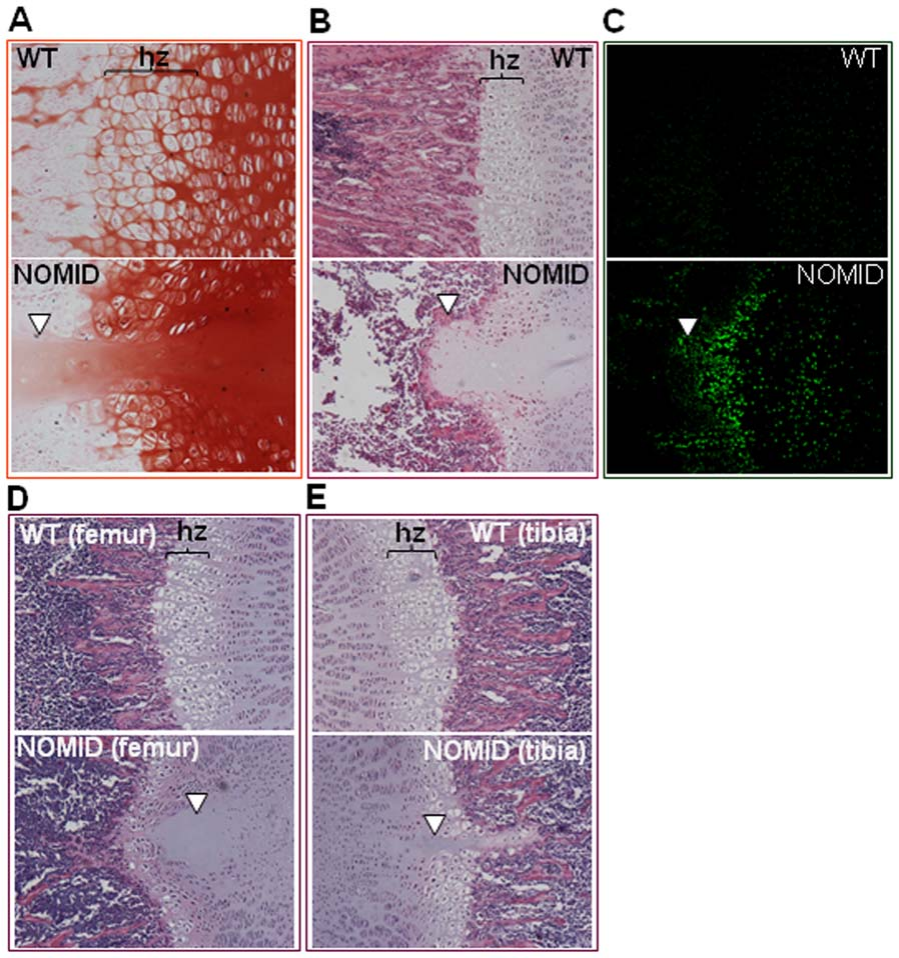
NOMID mice exhibit disorganized growth plates. Femoral sections from P13 (*A*–*C*) or P8 (*D* and *E*) mice were used for safranin O (*A*) and H&E (*B*, *D* and *E*) staining or for TUNEL (*C*). Original magnification: ×20 (*A* and *C*), ×10 (*B*, *D* and *E*). The spike (arrowhead) and early morphological changes (D, arrowhead) were observed only in NOMID mice. NOMID cells showed a high degree of apoptosis. hz, hypertrophic zone.

**Figure 5:**
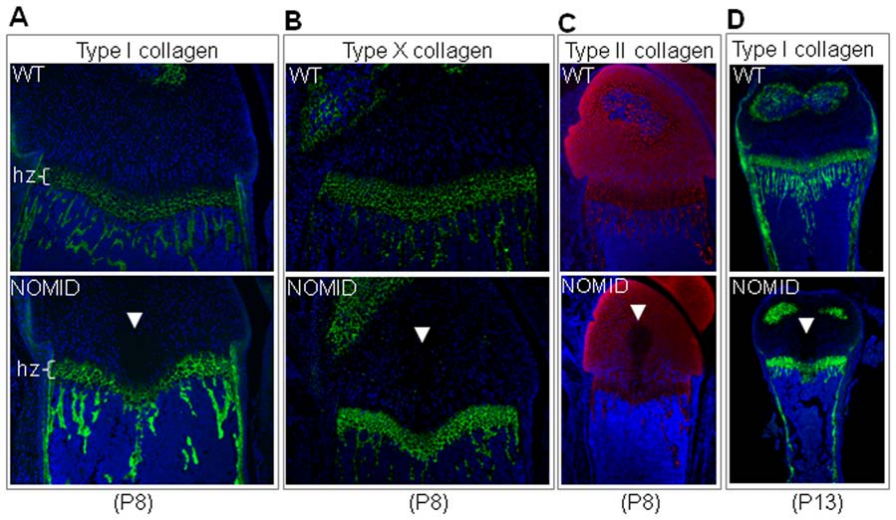
Type II collagen staining is reduced in the acellular structure in NOMID mice. Femoral sections from P8 (*A*, *B* and *C*) or P13 mice (*D*) were stained for types I, X (green), II (red) collagen, and counterstained with DAPI (blue). Type II collagen staining was not observed in the acellular structure within the cartilage zone above the hypertrophic chondrocyte zone (hz). Original magnification: ×20 (*A* and *B*), ×10 (*C* and *D*).

Histologic examination of the femurs from P13 mice confirmed the presence of the tissue spike in the center of the growth plate, extending into the primary spongiosa of the distal femur. The spike was acellular and stained in a gradient manner by the cartilage specific stain, safranin O (Fig. 4A). The thickness of the hypertrophic zone of the growth plate was reduced in NOMID compared to WT mice (Fig. 4A and 4B). Hypertrophic chondrocytes of NOMID mice showed a high degree of apoptosis, evidenced by TUNEL staining, particularly in the area surrounding the spike (Fig. 4C). Interestingly, H&E staining of P8 bones further revealed the presence of the acellular central structure in the epiphysis of the distal femur and proximal tibia (Fig. 4D and 4E). We observed the development of the spike in 12 out of 12 NOMID mice, but in 0/15 WT siblings. In P8 bones, the expression patterns of type I collagen and type X collagen, components of the extracellular matrices of bone and the hypertrophic zone, respectively, were indistinguishable between WT and NOMID mice, although the hypertrophic zone was thinner in NOMID especially in the mid region (Fig. 5A and 5B). Interestingly, the staining of type II collagen, which is selectively expressed by chondrocytes, was reduced in the central zone of the epiphysis, with a void corresponding to the acellular area (Fig. 5C). The region of the tissue spike below the growth plate stained positively for type I collagen, particularly in P13 femurs (Fig. 5D). Thus, safranin O gradient staining patterns coupled with type I collagen staining and the μCT evidence suggest that the spike contained elements of bone and cartilage matrices.

### NOMID mice develop inflammation in bone marrow resulting in increased bone resorption

**Figure 6:**
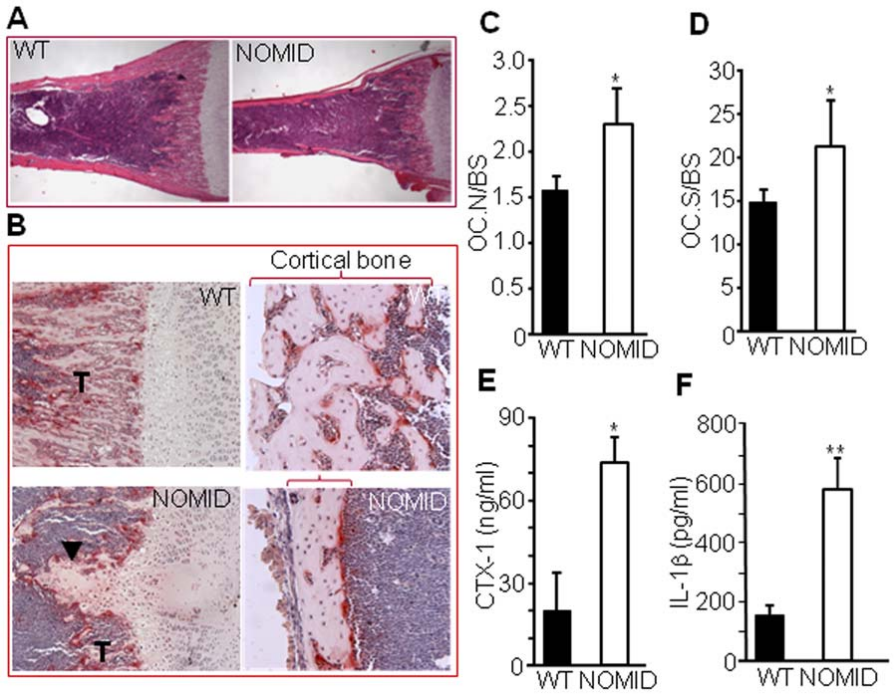
Bone resorption is increased in NOMID mice. Femoral sections from P13 mice were stained with H&E (*A*) or for TRAP activity (*B*–*D*), and OC number (*C*) or surface (*D*) per bone surface was determined. NOMID mice exhibited a larger bone marrow cavity in relation to overall bone size compared to WT mice (*A*). Abundant OC (stained in red, *B*) were present in the primary spongiosa and on the endocortical bone surface of NOMID mice. There were fewer trabeculae (T) and thinner cortical bone (bracket) in NOMID compared to WT mice. Original magnification: ×2 (*A*), ×10 (*B*, trabecular region), ×20 (*B*, cortical region). Serum was collected for the measurement of CTX-1 levels (*E*), and supernatants were collected from centrifuged bone marrow for the measurement of IL-1β (F). CTX-1 and IL-1β levels were 3- to 4-fold higher in NOMID compared to WT mice. Quantitative data were obtained from 5 mice/genotype and expressed as the mean ± S.D. (*C*, *D* and *F*) or mean ± S.E.M. (*E*). *P<0.05; **P<0.007.

The decreased trabecular bone volume (Fig. 3F) and cortical area and thickness (Fig. 3G and 3H) in NOMID mice may be the result of increased bone resorption, decreased bone formation or both. However, the disproportionately increased bone marrow cavity relative to the small bone size of NOMID mice (Fig. 6A) suggests increased bone resorption. Consistent with this notion, we observed abundant tartrate-resistant acid phosphatase (TRAP) positive OC in the primary spongiosa and on the endocortical bone surface of NOMID compared to WT mice (Fig. 6B). Quantitative analysis indicated significantly increased OC number (Fig. 6C) and OC surface (a reflection of OC size) (Fig. 6D) in NOMID mice, associated with a 4-fold increase in the serum levels of C-telopeptide of type I collagen (CTX-1), a marker of bone resorption, compared to WT mice (Fig. 6E).

**Figure 7:**
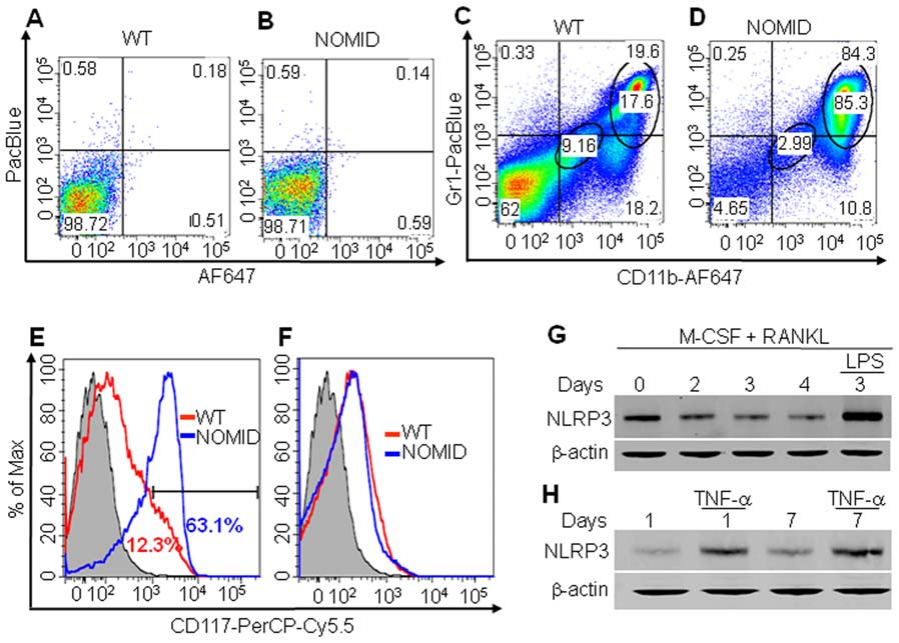
NOMID mice exhibit inflammation in the bone marrow, and bone cells express NLRP3. Bone marrow cells were unstained (*A* and *B*) or stained with antibodies against CD11b, Gr1 or CD117 (*C* and *D*). The expression of CD117 was analyzed by gating cells expressing low levels (*E*) or high levels (*F*) of CD11b and Gr1. The number of CD11b^low^/Gr1^low^/CD117^+^ cells, but not CD11b^high^/Gr1^high^/CD117^+^ cells, was increased in NOMID mice. Flow cytometry experiments were repeated up to 4 times with similar results. (*G*) BMM were induced to differentiate into OC in the presence of M-CSF and 100 ng/ml RANKL for the indicated times, and some cultures were further stimulated with 100 ng/ml LPS for 24 hours. (*H*) BMSC were induced to differentiate into OB for 1 or 7 days, and some cultures were then stimulated with 20 ng/ml TNF-α for 24 hours. Western blot analysis shows that NLRP3 expression was maintained throughout cell differentiation and was up-regulated by LPS or TNF-α. β-actin was used as a protein loading control.

Consistent with constitutively activated NLRP3, IL-1β levels in fluid obtained by centrifuging bone marrow were approximately 3-fold higher in NOMID compared to WT mice (Fig. 6F), indicative of inflammation in the bone marrow. Accordingly, FACS analysis revealed that the number of cells expressing neutrophil markers (CD11b^+^/Gr1^+^ or CD11b^+^/Ly6G^+^) or inflammatory monocyte markers (CD11b^+^/Ly6C^+^) was increased in NOMID bone marrow compared to WT (Fig. S5). Within these cell populations (Fig. 7C and 7D), the number of CD11b^low^/Gr1^low^/CD117^+^ cells, those with higher potential to form OC, was increased in NOMID bone marrow (Fig. 7E), but not CD11b^high^/Gr1^high^/CD117^+^ cells (Fig. 7F). These data are consistent with neutrophilia (Fig. 2A) and an increase in the number of OC in NOMID mice. Thus, inflammation-associated bone resorption accounts at least in part for the low bone mass of NOMID mice.

### NLRP3 inflammasome and its effectors are expressed by the OC and OB lineages

To delineate the expression profile of the NLRP3 inflammasome and its effectors in bone cells, we performed RT-PCR analyses on cDNA generated from OC and OB lineages. IL-1β and IL-18 messages were expressed by WT OC and OB lineages as was TLR-4 mRNA (Fig. S6). Importantly, Western blotting analysis revealed that NLRP3 protein expression was maintained throughout the differentiation of WT OC (Fig. 7G) and OB though its levels were slightly lower in OB (Fig. 7H). In addition, our results show up-regulation of NLRP3 expression in the OC and OB lineages by LPS (a TLR-4 ligand) and TNF-α, respectively. Thus, NLRP3 expression in OC and OB lineage cells is regulated by inflammatory stimuli *in vitro*.

### OC differentiation *in vitro* is increased in NOMID cells

**Figure 8:**
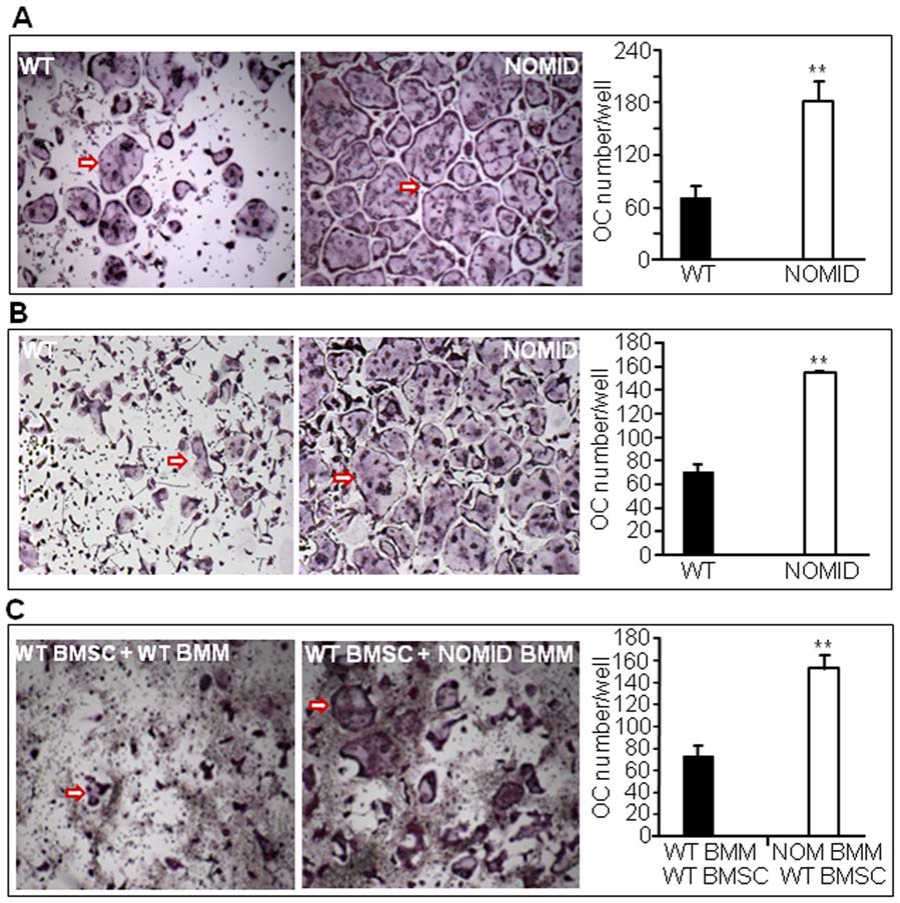
OC differentiation is increased in NOMID cells. Unfractionated bone marrow cells (*A*) or BMM previously cultured for 3 days in the presence of M-CSF (*B*) were induced to differentiate into OC in the presence of M-CSF and 100 ng/ml RANKL. (*C*) Co-cultures of WT or NOMID (NOM) BMM and WT BMSC were carried out in the presence of 10 nM dexamethasone and 1 nM 1,25(OH)_2_ vitamin D_3_ for 5–7 days. The cultures were stained for TRAP activity, and the number of OC (cells stained in red with ≥3 nuclei, arrow) were counted manually. The pictures were taken at the same magnification (×4) for both genotypes. The data show that NOMID cells formed more OC than WT cells. Determinations were performed in triplicate and expressed as the mean ± S.E.M. Results are representative of at least three independent experiments. **P<0.007.

Next, we studied OC differentiation *in vitro* from unfractionated bone marrow cells. We found that RANKL and M-CSF-stimulated OC differentiation was significantly higher in NOMID cells compared to WT (Fig. 8A). To determine whether this is due to an increase in the number of OC precursors, hyper-responsiveness of these cells to M-CSF and RANKL or a combination thereof, we studied OC formation using bone marrow-derived macrophages (BMM). NOMID BMM formed more OC than WT cells in response to RANKL and M-CSF treatment (Fig. 8B). Co-culturing NOMID BMM with WT bone marrow stromal cells (BMSC) also resulted in a significantly higher number of OC relative to co-cultures of WT BMM and WT BMSC (Fig. 8C), suggesting that NOMID BMM have a higher predisposition to differentiate into OC.

**Figure 9:**
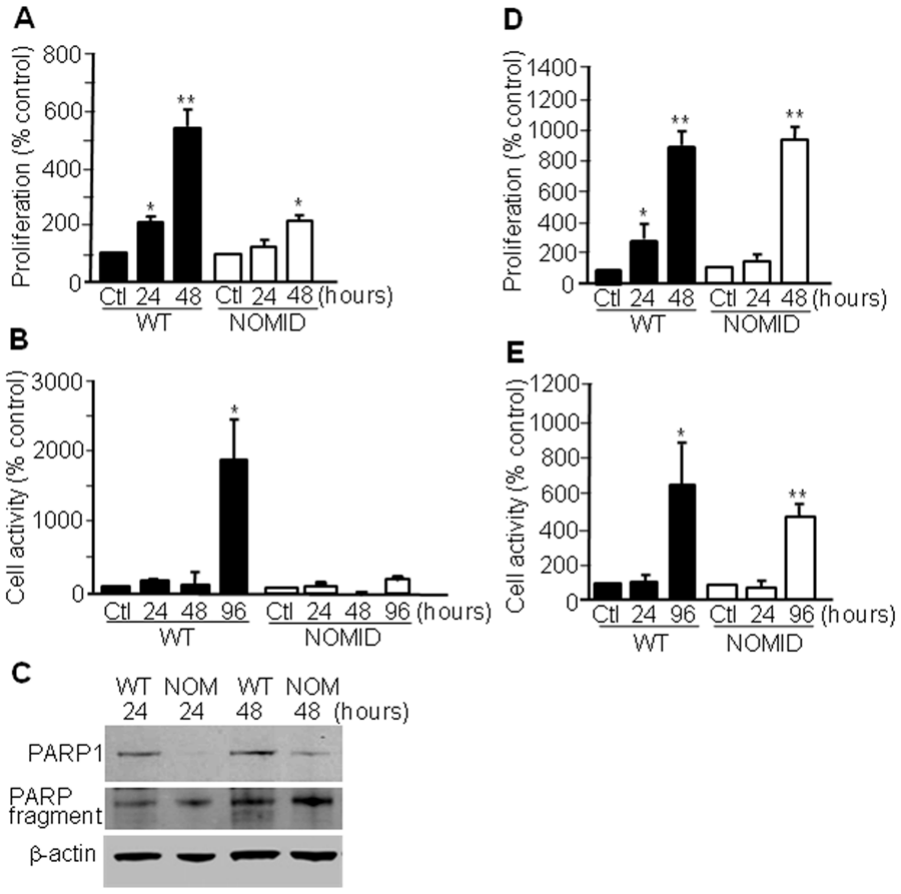
Unfractionated NOMID bone marrow cells proliferate and survive significantly less than their WT counterparts. Unfractionated bone marrow cells (*A*–*C*) or BMM (*D* and *E*) were cultured in media containing M-CSF for the indicated times. Proliferation (*A* and *D*), metabolic activity (*B* and *E*) and Western blot analysis of PARP cleavage (*C*) were carried out. While unfractionated NOMID cells proliferated and survived less than WT cells, no differences were seen in BMM proliferation and survival between genotypes. PARP cleavage was higher in NOMID than in WT cells. Determinations were performed in triplicate and expressed as the mean ± S.D. Results are representative of three independent experiments. *P<0.05; **P<0.007 over the control (Ctl).

Next, we determined the ability of OC precursors to respond to M-CSF-induced mitogenic and survival signals. We observed that unfractionated NOMID bone marrow cells proliferated significantly less in response to M-CSF than their WT counterparts (Fig. 9A), in agreement with a recent report indicating that OC precursors with higher osteoclastogenic potential are less responsive to the mitotic effect of M-CSF [22]. Within a 96-hour timeframe, cell metabolic activity (a reflection of cell proliferation and survival) was diminished in NOMID cells compared to WT (Fig. 9B), consistent with the increased levels of cleaved PARP, a marker of apoptosis, in NOMID cells (Fig. 9C). The overall decrease in cell survival may reflect neutrophil loss *in vitro* as these cells are expected to survive poorly in such experimental conditions. Interestingly, no difference in cell proliferation and survival between genotypes was observed when cultured BMM were used (Fig. 9D and 9E).

It is possible that the increased osteoclastogenesis in NOMID cells is due to increased activation of key pathways regulating OC development. To test this hypothesis, we performed Western blot analyze the activation of NF-κB, Akt, ERK and JNK pathways in NOMID and WT cells. M-CSF caused a similar degree of ERK and Akt phosphorylation in WT and NOMID BMM (Fig. S7A), findings consistent with the mitogenic effects of M-CSF on these cells. We also found that RANKL-mediated activation of NF-κB, ERK and JNK pathways was comparable between WT and NOMID BMM (Fig. S7B). Collectively, these findings support the hypothesis that the NOMID mutation causes increased osteoclastogenesis *in vitro* independent of hyper-activation of MAPK, NF-κB and Akt pathways.

## Discussion

The identification of more than 80 disease-associated mutations in *NLRP3* in patients with CAPS underscores the challenges of genotype-phenotype relationship studies in humans [8], [9]. In an attempt to generate pre-clinical disease-relevant models and investigate the phenotypic features unique to NOMID including the characteristic arthropathy and severe CNS sequelae, we engineered mice expressing a D301N NLRP3 mutation, the mouse ortholog of a human mutation associated with the most severe CAPS phenotype, NOMID. NOMID mice were growth retarded compared to WT littermates and developed systemic inflammation as evidenced by leukocytosis and increased levels of multiple inflammatory mediators, demonstrating that D301N is a gain-of-function mutation as anticipated. In agreement with previous reports, cytokines associated with T cell activity were not up-regulated, providing further evidence for the CAPS paradigm of fully innate immune driven inflammation [20]. The similarities in cell types, cytokine profiles, and overall gross phenotype suggest these mice are reasonable models of human NOMID. While the CNS phenotype of these mice is limited, we did identify a striking bone phenotype that is the focus of this current study.

Histologic examination of mouse knee joints revealed neutrophilic infiltration in the bone marrow, synovium and surrounding tissue, indicating potential skeletal inflammation. Subsequent analysis of P13 femurs and tibiae showed severely disorganized growth plates associated with the development of a tissue spike across the mid region of the growth plate in all NOMID mice, but not WT controls. Most cells inside and around this structure were apoptotic, an observation in agreement with its acellularity and reduced type II collagen staining. Morphologic changes in NOMID bones were mostly confined to the center of the epiphysis at P8. Taken together, these results suggest that increased chondrocyte apoptosis in the center of the growth plate resulted in the spike formation. Further studies on the role of NLRP3 mutations on chondrocyte homeostasis are needed and will be performed by taking advantage of a chondrocyte-specific Cre recombinase system to limit expression of D301N NLRP3 to cells involved in skeletal homeostasis. By side-stepping the severe systemic inflammation caused by universal D301N NLRP3 expression, these mutant mice are likely to live longer, enabling long-term studies.

The tissue spike developed disproportionally in the knee, as its size was larger in the femoral distal epiphysis compared to the tibial proximal epiphysis. We therefore propose that the tissue spike could ultimately lead to the development of asymmetric knee deformations as seen in NOMID patients. Interestingly, abnormal endochondral bone formation in these patients has been suggested, although the histologic analysis was limited to a single biopsy specimen of the growth plate of the distal femoral epiphysis from one patient [12]. Whether the spike could result in classic NOMID arthropathy unfortunately could not be determined within the limited life span of these mice.

We show that the D301N NOMID mutation causes massive bone resorption, echoing the osteoporosis commonly observed in NOMID patients [12]. The osteopenia noted in NOMID mice likely occurred through multiple mechanisms. Indeed, our studies suggest that the NOMID mutation results in an increased number of bone marrow OC precursors, perhaps linked to the high levels of pro-osteoclastogenic cytokines in the bone marrow milieu, but also heightened sensitivity to osteoclastogenic signals, as demonstrated by the significantly increased number of OC produced *in vitro* by NOMID BMM co-cultured with WT BMSC. The molecular mechanisms of this increased osteoclastogenesis remain to be further elucidated not only because we found no difference in the ability of M-CSF or RANKL to activate several signaling pathways between NOMID and WT cells, but also because skeletal lesions in NOMID patients continue despite IL-1β blockade therapies [23]. In any case, it is likely that this dramatically increased osteoclastogenesis accounts for most of the reduced trabecular bone mass and cortical thickness present in our NOMID mice. It is possible, however, that decreased bone formation also contributed to the osteopenic phenotype. OB differentiation studies were hampered by the poor growth of NOMID bone-forming cells *in vitro* and OB-related bone parameters could not be obtained from a P13 immature skeleton.

It is noteworthy that NOMID mice are clearly unhealthy. Therefore, the skeletal analysis of these mice may be confounded by a number of factors including low body weight and systemic inflammation. The development of the tissue spike as a nonspecific consequence of systemic inflammation cannot be ruled out since these mice globally express mutant NLRP3, however to the best of our knowledge, tissue spike has never been reported in any of the various models of skeletal pathologies associated with systemic inflammation, such as the TNF trangenic model [24]. Mice deficient in the phosphatidylinositol 3′(IP3)-phosphatase, Pten have been shown to develop a bony “bridge" [25] structure that superficially resembles the NOMID tissue spike. However, Pten deficient mice had no evidence of inflammation and actually exhibited higher bone mass than controls. No relationship between the Pten pathway and the inflammasome has been reported.

Irrespective of the underlying mechanisms, our findings provide some insights on the mechanisms of NOMID-related arthropathy, which is prominent in the knees and is characterized by large epiphyseal expansion. Whether the skeletal phenotype is truly specific to the NOMID mouse, and not found in other *Nlrp3* mutant models cannot currently be determined, as mice expressing MWS and FCAS mutations die even earlier than their NOMID counterparts [20]. Thus, leveraging strategies confining mutant NLRP3 expression to certain cell types could further differentiate mouse mutant NLRP3 phenotypes. Our findings do indeed establish the validity of this mouse NOMID model and establish the NLRP3 inflammasome as a major regulator of pathological bone resorption, opening novel avenues for future investigations on the mechanisms underlying the skeletal manifestations associated with NOMID.

## Materials and Methods

### Generation of NOMID mice

The D301N allele was engineered by site directed mutagenesis with a Stratagene Quik-change kit using a previously described strategy [20]. Briefly, the targeting construct pPNTlox2PNlrp3D301N was created by cloning 4–7 kb regions directly upstream and downstream of a targeted position in intron 2 of *Nlrp3* around the *neoR* antibiotic resistance cassette in plasmid pPNTlox2P (Fig. S1). 129 SvJ stem cells were electroporated with linearized pPNTlox2PNlrp3D301N as described previously [20]. Stem cell colonies were selected based upon Southern analysis and PCR and used to create chimeric mice, which yielded offspring heterozygous for D301N. Zona pelucida 3-Cre recombinase-mediated excision of the *neoR* cassette was confirmed, and mouse genotyping were performed with PCR. All procedures were approved by the Animal Studies Committee of the University of California, San Diego (UCSD), La Jolla, and Washington University in St. Louis.

### Bone mass and microstructure

Whole BMD and femoral bone structure were analyzed by DXA using a PIXImus scanner (GE/Lunar, Madison, WI) and μCT system (μCT 40; Scanco Medical AG, Zurich, Switzerland), respectively, as previously described [26]. For X-ray radiography, animals were anesthetized, placed in prone and lateral positions against the Biomax XAR film (Kodak Scientific Imaging), and exposed to an X-ray at 20 kV for 15 sec using a Faxitron radiographic system (Faxitron X-ray Corporation). Films were developed using a Kodak X-OMAT 2000A processor. Micro-Ct scans were derived as described previously [26]. Briefly, femora were stabilized in 2% agarose gel and μCT scans taken along the length of the femur (16 µm voxel size, 16.4 mm diameter, 45 kVp, 145 µA, medium resolution, and 150 ms integration time). The volume of interest analyzed was located just distal to the physis, spanning a height of 700 µm each for the metaphyseal region containing the spike and contiguous to this structure, and contained all the bone within the cortical shell (Fig. 3D).

### Histology, histomorphometry and immunofluorescence

Tissue samples were processed as described previously. Briefly, bones and brains were fixed in 10% formalin. Bones decalcified in 14% (w/v) EDTA pH 7.2 for 10–14 days at room temperature, embedded in paraffin, sectioned at 5 µM thickness were mounted on glass slides. The sections were stained with H&E, TRAP or safranin O as previously described [27]. Apoptosis was analyzed using the fluorescein In Situ Cell Death Detection kit (Roche). For immunofluorescence, P8 limb tissue sections were incubated with 1% hyaluronidase (Sigma) for 30 min at 37°C, rinsed with PBS and blocked with 10% goat serum for 1 hour at room temperature. The sections were then incubated overnight at 4°C with rat polyclonal type II collagen antisera [28], rabbit polyclonal type I collagen antisera (Abcam) or rabbit polyclonal type X collagen antisera [29]. After washes in PBS, the sections were incubated for 1 hour at room temperature with secondary antibodies conjugated to either Alexa 488 or Alexa 594 fluorescent dyes (Invitrogen). Following rinses in distilled water, DAPI-containing mounting solution was applied to each tissue section. A Nikon Eclipse E800 fluorescence microscope was used to view the images.

### Peripheral blood analysis

Complete blood counts were performed by the UCSD ACP Diagnostic Laboratory as previously described [20].

### Preparation of bone marrow cells

The femora and tibiae were harvested into α-MEM containing 10% FBS (Invitrogen), 100 µg/ml streptomycin and 100 IU/ml penicillin G (culture media, CellGro) after removal of excess tissues. Bone marrow cells were eluted from the bone marrow cavity by first removing the epiphyses of bones followed by centrifugation at 13,000 rpm for 30 sec. Cells were then resuspended in culture medium and filtered to remove debris.

### Osteoclast (OC) formation

For OC formation from unfractionated bone marrow cells, cells (5×10^4^) were plated in a 96-well plate in culture media containing a 1∶50 dilution of CMG 14–12 supernatant as a source of M-CSF and RANKL (100 ng/ml) [30]. Media with supplements were changed every other day for 5–7 days as indicated, and maintained at 37°C in a humidified atmosphere of 5% CO_2_ in air.

For OC formation from enriched BMM, bone marrow cells were maintained in culture media containing a 1∶25 dilution of CMG 14–12 supernatant as a source of M-CSF for 5 days in a 10-cm dish. Nonadherent cells were removed by vigorous washes with PBS, and adherent BMM were detached with trypsin-EDTA, plated at 5–10×10^3^/well in a 96-well plate, and cultured as described above.

For co-cultures, BMSC were culture expanded in α-MEM containing 20% FBS (ATLAS) for 5–7 days. BMSC (37.5×10^3^) and BMM (37.5×10^3^) were plated per well in a 96-well plate in α-MEM supplemented with 10 nM dexamethasone and 1 nM 1,25(OH)_2_ vitamin D_3_ (Sigma) as previously described [31]. Cells were cultured as described above.

### Osteoblast (OB) differentiation

BMSC were induced to differentiate into OB as previously described [26]. Briefly, BMSC (8×10^5^) were plated per well in a 6-well plate in α-MEM supplemented with osteogenic medium containing 10 mM β-glycerophosphate and 50 µg/ml ascorbic acid.

### Flow cytometry

Mouse bone marrow cells were prepared as described above. Red blood cells were depleted with red blood cell lysis buffer (Roche), and fixed in PBS containing 1% bovine serum albumin and 0.1% sodium azide. Cells (0.5–1×10^6^) were incubated with CD16/32 (BioLegend) to block nonspecific Fc binding, stained with PE-anti-Ly6G monoclonal antibodies (mAbs), Alexa-Fluor®647-anti-CD11b mAbs, PerCP-Cy™5.5-anti-CD117 mAbs (BioLegend), PerCP-Cy™5.5-anti-Ly6C mAbs or V450-anti-Gr1 mAbs (BD Pharmingen), according to the supplier's instructions. Samples were acquired with a FACSCaliber (BD) or FACSCanto (BD), followed by analysis with FlowJo software (Tree Star, Inc.).

### Cell proliferation and metabolic activity

Mouse bone marrow cells (5×10^4^) prepared as described above were plated in a 96-well plate and treated with M-CSF-containing supernatant for the indicated times. Proliferation and metabolic activity were analyzed using the BrdU chemiluminescent ELISA kit (Roche) and the MTT method (Sigma), respectively.

### TRAP staining

Cytochemical staining for TRAP was used to identify OC as described previously [27], [28], [32]. Briefly, cells in a 96 well plate were fixed with 3.7% formaldehyde and 0.1% Triton X-100 for 10 min at room temperature. The cells were rinsed with water and incubated with the TRAP staining solution (Sigma leukocyte acid phosphatase kit) at room temperature for 30 min. Multinuclear TRAP positive cells with at least 3 nuclei were counted under light microscopy.

### Western blot analysis

Mouse BMM (8×10^5^) were plated per well in a 6-well plate in media containing 20 ng/ml M-CSF and/or 100 ng/ml RANKL, and cultured for the indicated times. Some cultures were treated with 100 ng/ml LPS (Sigma) for 24 hours. Mouse BMSC (1×10^6^) were plated per well in a 6-well plate in osteogenic media containing 10 mM β-glycerophosphate and 5 µg/ml ascorbic acid for 7 days. At day 1 or day 7, cells were treated with 20 ng/ml of mouse TNF-α (R&D Systems) for 24 hours. Cell extracts were prepared by lysing cells with RIPA buffer (50 mM Tris, 150 mM NaCl, 1 mM EDTA, 0.5% NaDOAc, 0.1% SDS, 1.0% NP-40) plus phosphatase inhibitors (2 mM NaVO4, 10 mM NaF, 1 mM PMSF) and Complete Protease Inhibitor Cocktail (Roche). Protein concentrations were determined by the BioRad method, and equal amounts of proteins were subjected to SDS-PAGE on 4–12% NuPAGE gels (Invitrogen). Proteins were transferred onto nitrocellulose membranes, and incubated with antibodies against NLRP3 (Adipogen), p-cJun (Santa Cruz Biotechnology), p-ERK and p-Akt (Cell Signaling) or β-actin (Sigma) for 2 hours at room temperature, followed by 1 hour incubation with secondary goat anti-mouse IRDye 800 (Rockland) or goat anti-rabbit Alexa-Fluor 680 (Molecular Probes), respectively. The results were visualized using Li-Cor Odyssey Infrared Imaging System (Li-Cor).

### Immunoassays

Serum cytokine levels were analyzed by Luminex assay (Bio-Rad) according to the manufacturer's instructions. Serum CTX-1 levels were quantified using a Rat LAPS EIA kit from Immunodiagnosticssystems. Bone marrow IL-1β levels were quantified using the eBiosciences Elisa kit.

### Statistical analysis

Statistical significance was assessed by Student's t test for independent samples. Values are expressed as mean ± S.E.M. unless otherwise stated.

## Supporting Information

Figure S1 **Generation of the D301N NLRP3 mutant.** The mutant allele was generated as described in Materials and Methods. The asterisk depicts the mutation in exon 3.
(TIF)

Figure S2 **Evidence of meningitis in NOMID mice.** Brains from P13 mice were stained with H&E. NOMID mice (*B*) showed a perivascular accumulation of neutrophils (arrows) in the meninges that is absent in WT mice (*A*).
(TIF)

Figure S3 Expression of inflammatory mediators in mouse serum. Serum cytokine analysis (5–7 mice/genotype) was carried out on samples harvested from P12–13 mice. Data are expressed as the mean ± S.E.M. *P<0.05; **P<0.007.
(TIF)

Figure S4 **Bone parameters in NOMID and WT mice.** Femoral metaphyseal region that included (*A*, *C* and *E*) or extended approximately 700 µm contiguous to the spike (*B*, *D* and *F*) were analyzed by μCT. Quantitative data were obtained from 5–6 mice/genotype. Trabecular number (Tb.N) and thickness (Tb.Th) were decreased, and trabecular space (Tb.Sp) was increased in NOMID mice in the region that is contiguous to the spike, but not in the region that contains this structure. Data are expressed as the mean ± S.E.M. *P<0.05; **P<0.007.
(TIF)

Figure S5 **Flow cytometry analysis of bone marrow cells.** Bone marrow cells were stained with antibodies against CD11b or Gr1 (*A* and *B*), CD11b or Ly6G (*C* and *D*), CD11b or Ly6C (*E* and *F*). The number of CD11b^+^/Gr1^+^ cells, CD11b^+^/Ly6C^+^ cells or CD11b^+^/Ly6G^+^ cells were approximately 3-fold higher in NOMID than in WT cells.
(TIF)

Figure S6 **Expression of IL-1 family members and TLR4 in bone cells.** Bones were harvested from WT or NOMID mice. RNA was isolated from flushed bone marrow cells (marrow, *A* and *D*), bone marrow-free bones (bone, *A*, *B* and *D*), BMSC induced or not to differentiate into OB for 2 weeks *in vitro* (OB, *C* and *E*) or BMM induced or not to differentiate into OC *in vitro* for 2 days in the presence of M-CSF and RANKL (pre-OC, *C*). RNA expression was analyzed by PCR. Cells of the OB or OC lineage expressed IL-1β, IL-18 and TLR4 transcripts.
(TIF)

Figure S7 **Activation of signaling pathways by M-CSF or RANKL in BMM.** BMM were treated with 50 ng/ml M-CSF (*A*) or 100 ng/ml RANKL (*B*), and proteins were analyzed by Western blot. M-CSF-mediated activation of ERK and Akt pathways or RANKL-mediated activation of ERK, NF-κB and JNK pathways was comparable between WT and NOMID BMM. The β-actin band shows that proteins were loaded equally. NS, non specific.
(TIF)
