# Supplementary figures and images for: Constitutively Activated NLRP3 Inflammasome Causes Inflammation and Abnormal Skeletal Development in Mice
Source: PLoS One. 2012 Apr 27;7(4):e35979. doi: 10.1371/journal.pone.0035979 (PMC3338787; doi:10.1371/journal.pone.0035979)

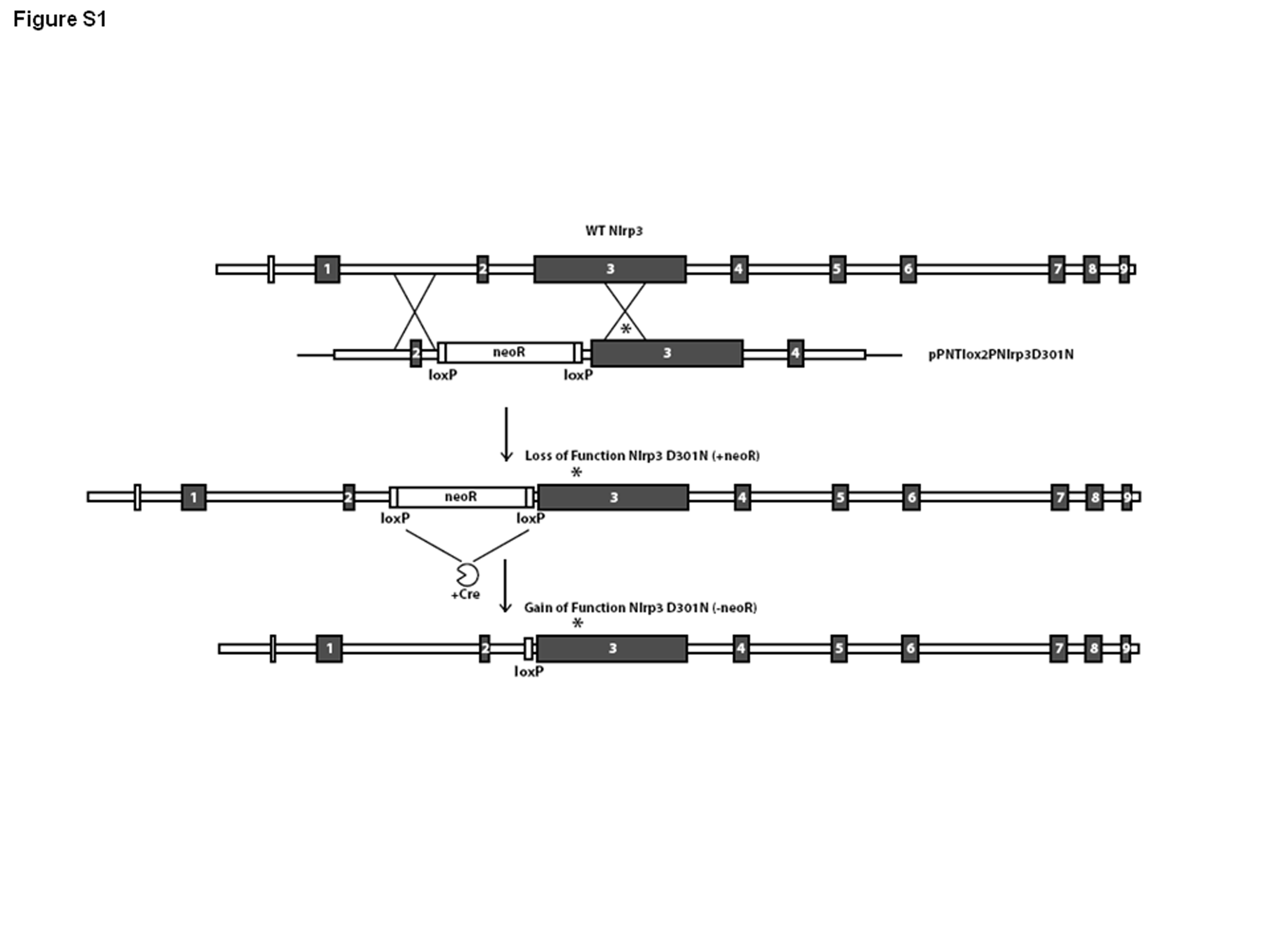

Supplement: Figure S1 — Generation of the D301N NLRP3 mutant. The mutant allele was generated as described in Materials and Methods. The asterisk depicts the mutation in exon 3. (TIF) [file pone.0035979.s001.tif]

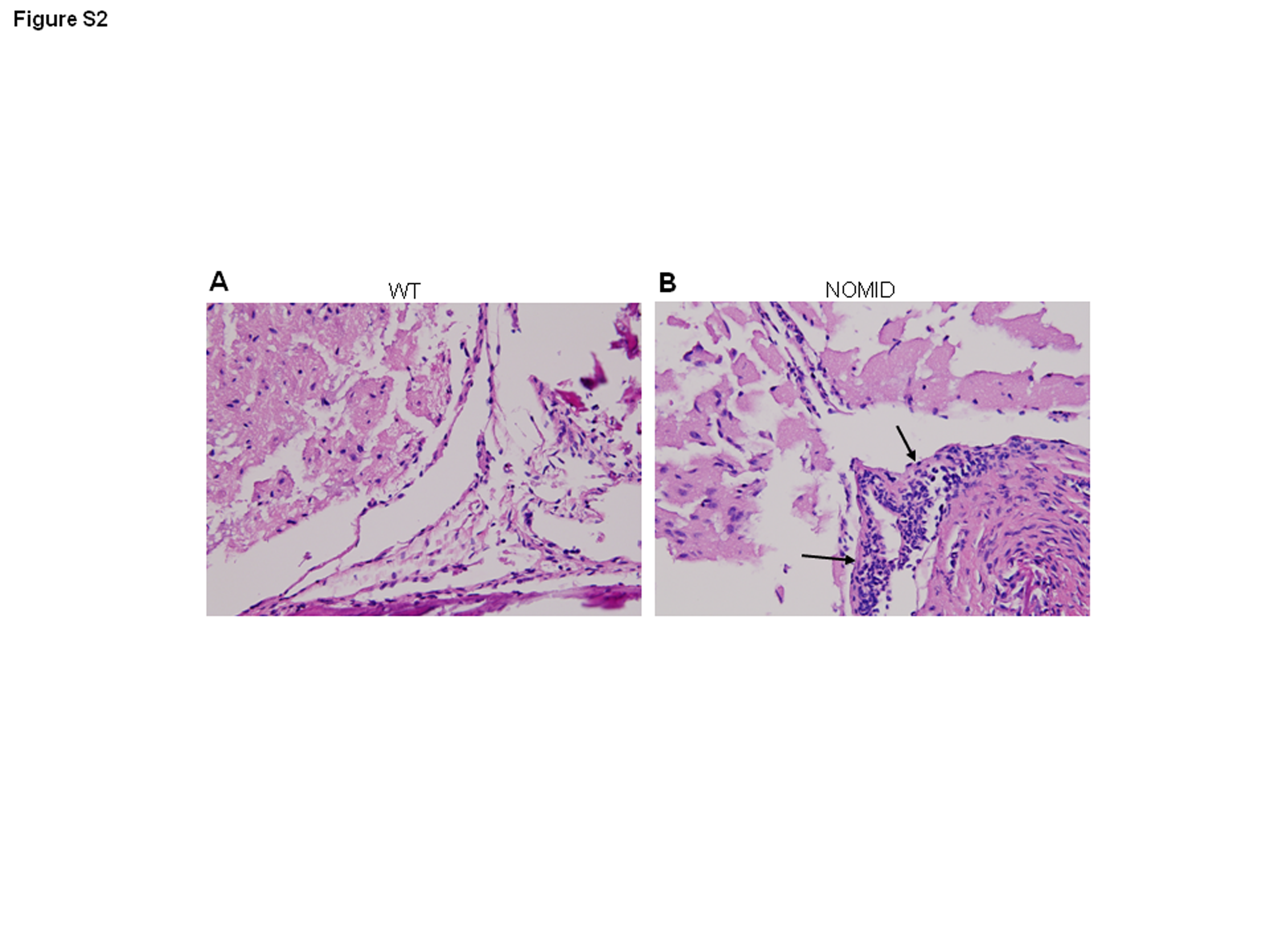

Supplement: Figure S2 — Evidence of meningitis in NOMID mice. Brains from P13 mice were stained with H&E. NOMID mice (B) showed a perivascular accumulation of neutrophils (arrows) in the meninges that is absent in WT mice (A). (TIF) [file pone.0035979.s002.tif]

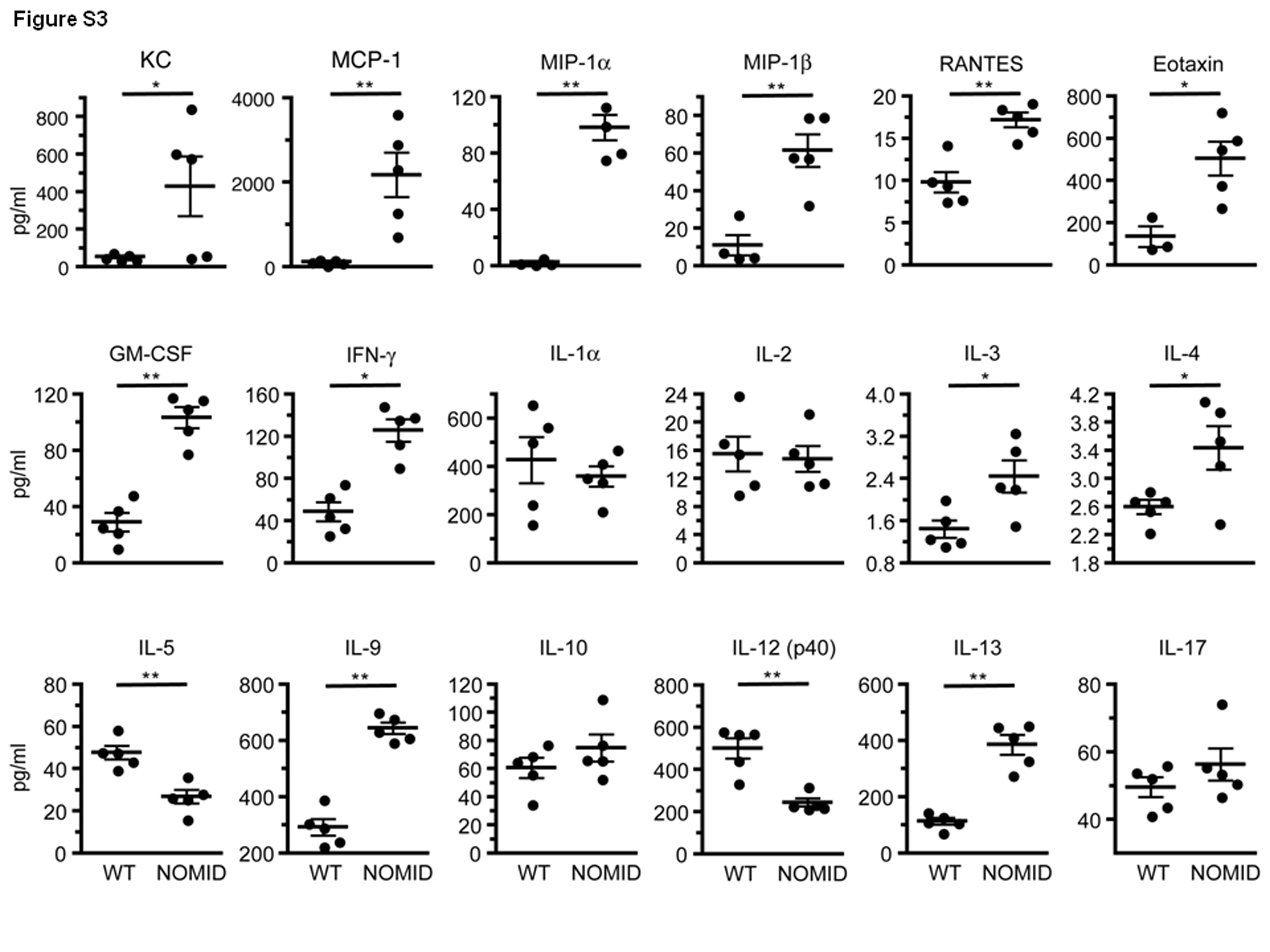

Supplement: Figure S3 — Expression of inflammatory mediators in mouse serum. Serum cytokine analysis (5–7 mice/genotype) was carried out on samples harvested from P12–13 mice. Data are expressed as the mean ± S.E.M. *P<0.05; **P<0.007. (TIF) [file pone.0035979.s003.tif]

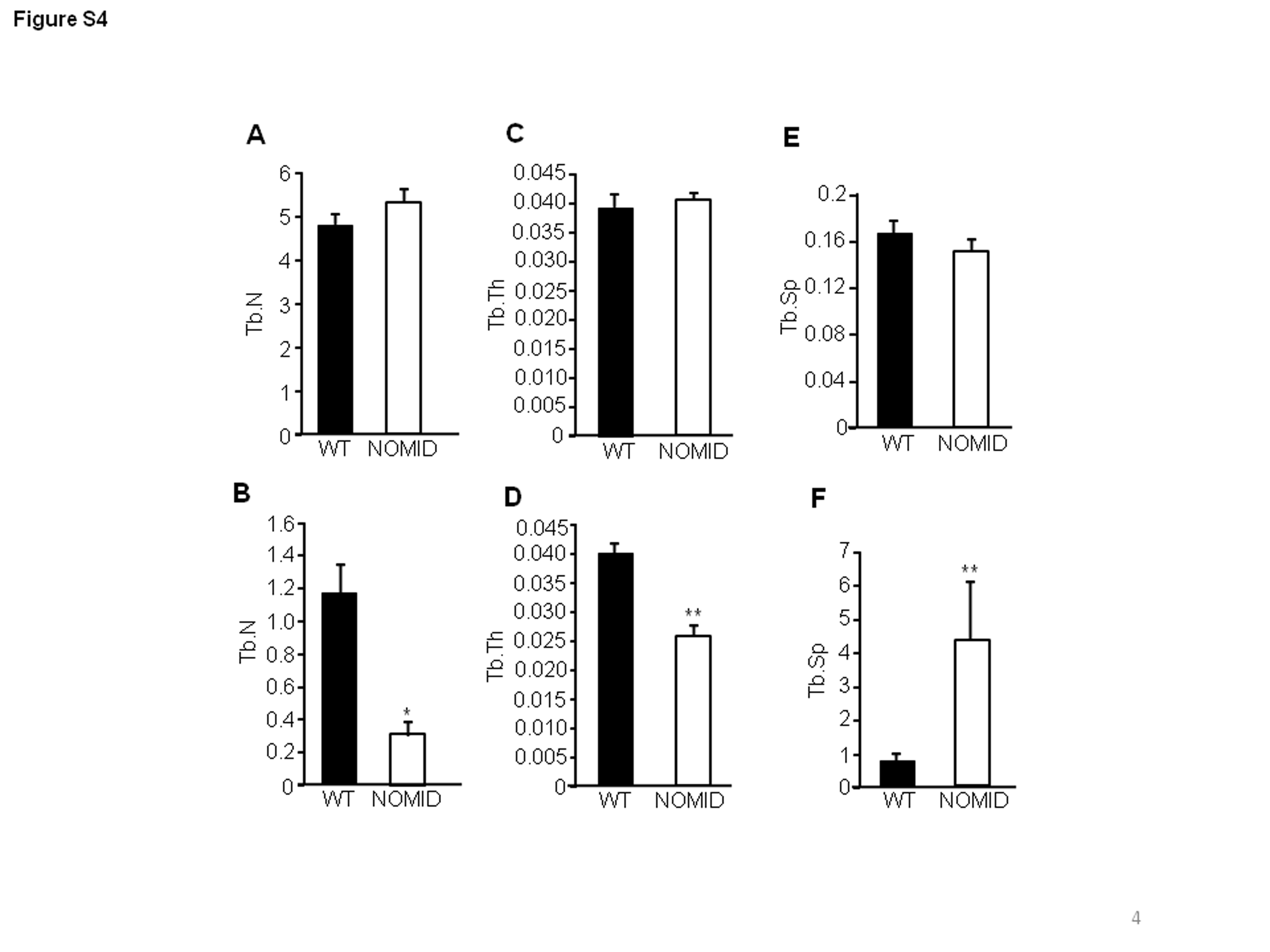

Supplement: Figure S4 — Bone parameters in NOMID and WT mice. Femoral metaphyseal region that included (A, C and E) or extended approximately 700 µm contiguous to the spike (B, D and F) were analyzed by μCT. Quantitative data were obtained from 5–6 mice/genotype. Trabecular number (Tb.N) and thickness (Tb.Th) were decreased, and trabecular space (Tb.Sp) was increased in NOMID mice in the region that is contiguous to the spike, but not in the region that contains this structure. Data are expressed as the mean ± S.E.M. *P<0.05; **P<0.007. (TIF) [file pone.0035979.s004.tif]

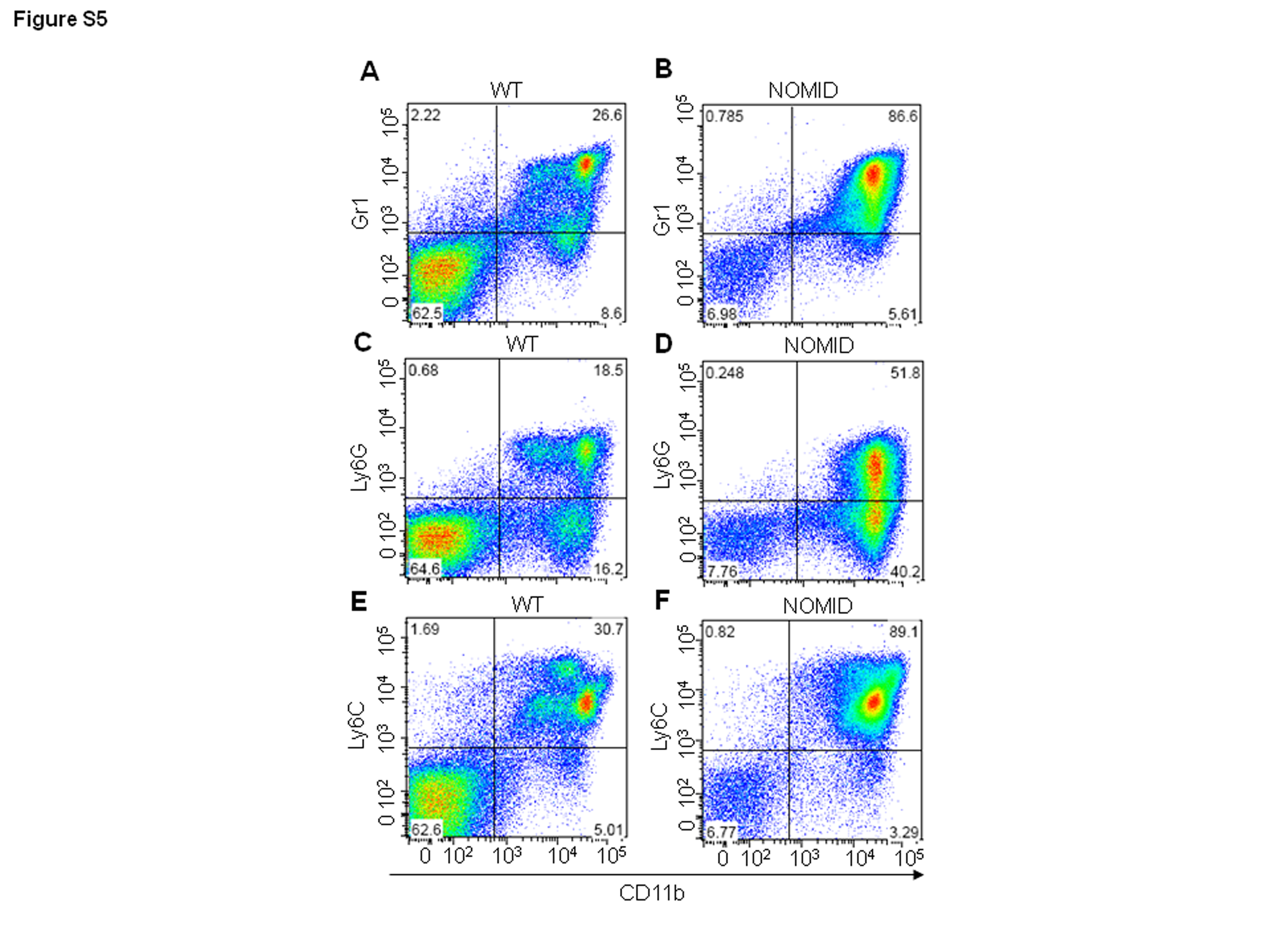

Supplement: Figure S5 — Flow cytometry analysis of bone marrow cells. Bone marrow cells were stained with antibodies against CD11b or Gr1 (A and B), CD11b or Ly6G (C and D), CD11b or Ly6C (E and F). The number of CD11b+/Gr1+ cells, CD11b+/Ly6C+ cells or CD11b+/Ly6G+ cells were approximately 3-fold higher in NOMID than in WT cells. (TIF) [file pone.0035979.s005.tif]

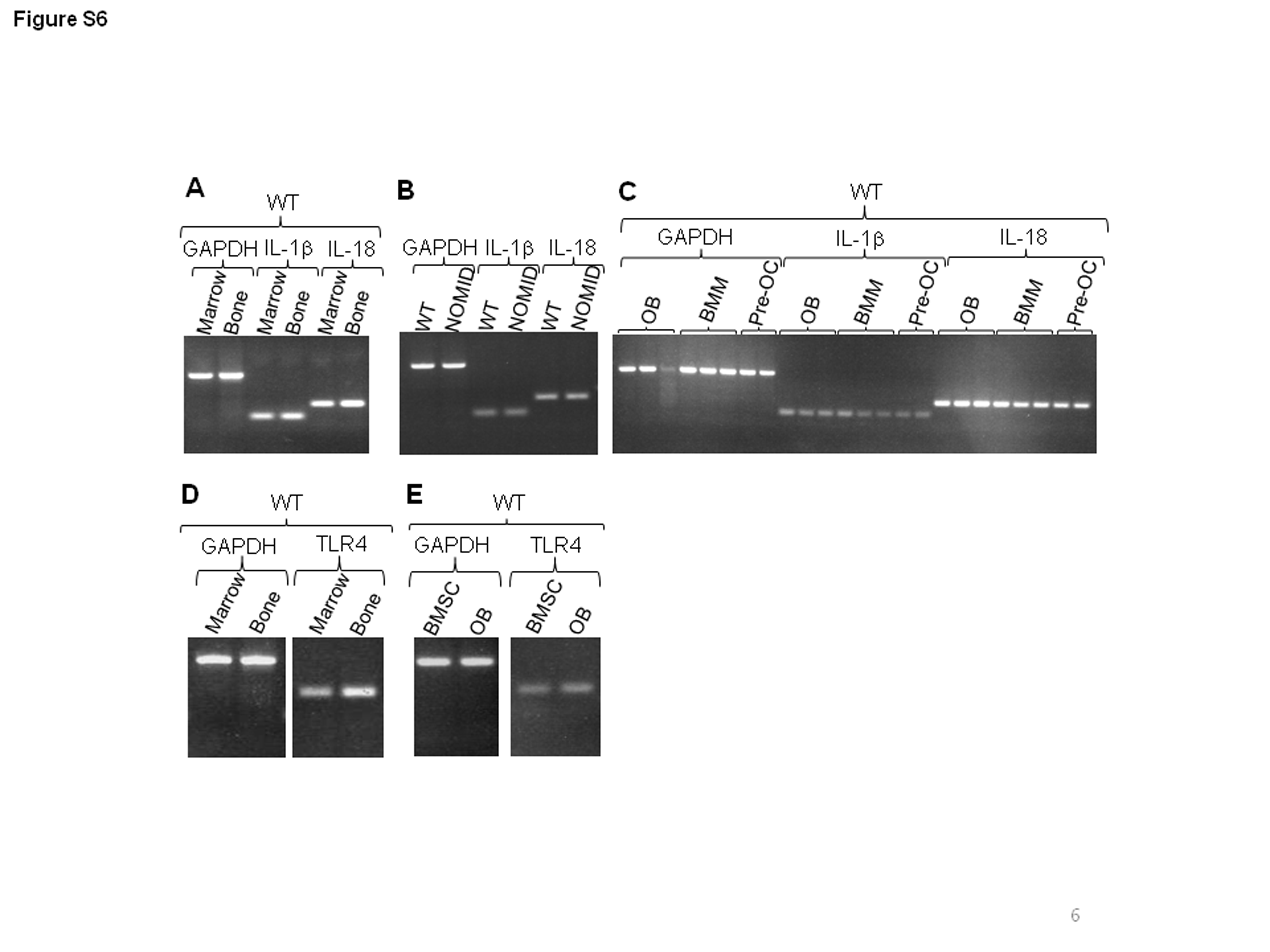

Supplement: Figure S6 — Expression of IL-1 family members and TLR4 in bone cells. Bones were harvested from WT or NOMID mice. RNA was isolated from flushed bone marrow cells (marrow, A and D), bone marrow-free bones (bone, A, B and D), BMSC induced or not to differentiate into OB for 2 weeks in vitro (OB, C and E) or BMM induced or not to differentiate into OC in vitro for 2 days in the presence of M-CSF and RANKL (pre-OC, C). RNA expression was analyzed by PCR. Cells of the OB or OC lineage expressed IL-1β, IL-18 and TLR4 transcripts. (TIF) [file pone.0035979.s006.tif]

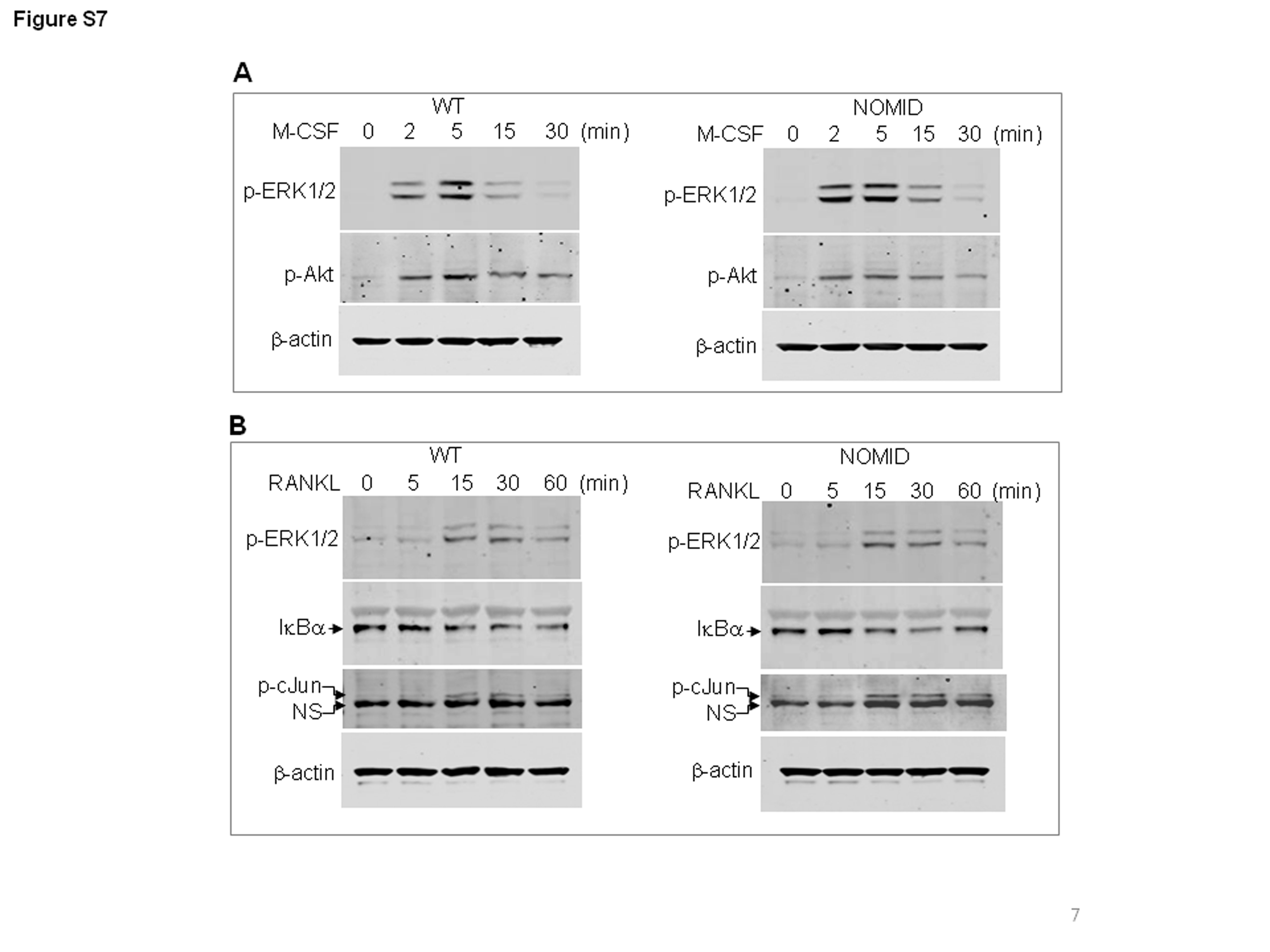

Supplement: Figure S7 — Activation of signaling pathways by M-CSF or RANKL in BMM. BMM were treated with 50 ng/ml M-CSF (A) or 100 ng/ml RANKL (B), and proteins were analyzed by Western blot. M-CSF-mediated activation of ERK and Akt pathways or RANKL-mediated activation of ERK, NF-κB and JNK pathways was comparable between WT and NOMID BMM. The β-actin band shows that proteins were loaded equally. NS, non specific. (TIF) [file pone.0035979.s007.tif]
